# Personalized alignment™ for total knee arthroplasty using the ROSA^®^ Knee and Persona^®^ knee systems: Surgical technique

**DOI:** 10.3389/fsurg.2022.1098504

**Published:** 2023-01-10

**Authors:** Vincent Massé, Jason Cholewa, Maged Shahin

**Affiliations:** ^1^ Hôpital Maisonneuve-Rosemont, Surgery Department, Université de Montréal, Montreal, Quebec, Canada; ^2^ Personalized Arthroplasty Society, Atlanta, GA, United States; ^3^ Duval Orthopaedic Clinic, Laval, Québec, Canada; ^4^ Zimmer Biomet, Warsaw, IN, United States

**Keywords:** kinematic alignment, orthopedic technology, restricted kinematic alignment, total knee arthroplasty, robotic assisted, rosa, personalized alignment

## Abstract

Total knee arthroplasty (TKA) procedures are expected to increase up to 565% in the United States over the next 3 decades. TKAs were traditionally performed with neutral mechanical alignments that provided equal medial and lateral gaps in extension and flexion to reduce implant wear but were less successful at restoring native knee function and associated with high patient dissatisfaction. Kinematic alignment (KA) restores native anatomy and minimizes soft tissue release; however, KAs that recreate severe deformities and/or biomechanically inferior alignments result in significant increases in implant stress and risk of aseptic loosening. Restricted kinematic alignment (rKA) recreates pre-arthritic anatomy within a range of acceptable alignment boundaries, and improved patient clinical scores and faster recoveries have been reported with rKA techniques. Personalized Alignment™ is an evolution of rKA that relies heavily upon robotic assistance to reliably recreate patient anatomy, native soft tissue laxity, and accurate component placement to improve patients' clinical outcomes. The purpose of this surgical technique report is to describe the Personalized Alignment TKA method using the ROSA^®^ Knee System and Persona^®^ The Personalized Knee^®^ implants. Herein we provide specific procedures for pre-operative planning, anatomical landmarking and evaluation, intra-operative planning and adjustment of resections and cuts, cut validation and soft tissue evaluation with robotic-assisted personalized TKA.

## Introduction

Total knee arthroplasty (TKA) is one of the most performed and effective musculoskeletal surgeries for treating osteoarthritis. In the United States alone, over 700,000 procedures are performed annually (1), and this value is projected to increase up to 565% by 2050 (2). Despite advances in component technology, patient dissatisfaction with TKA remains as high as 15%–20% (3, 4), with poor alignment and surgical variability thought to contribute to the pain, instability and range of motion limitations reported during activities of daily living following less successful TKAs (3, 5, 6).

The traditional objectives of TKA were to obtain a neutral alignment whereby components are implanted at 90° to the tibial and femoral mechanical axis (mechanical alignment: MA) with equal medial and lateral gaps in extension and flexion achieved through ligamentous release (7). While achieving a neutral mechanical axis and ligament isometry was once thought to increase implant survival, it does not appear to fully restore knee function (8, 9). In the native knee with intact cruciate ligaments, the flexion gap tends to be slightly wider than the extension gap, and greater lateral laxity is seen in flexion compared to extension (10–12). Kinematic alignment (KA) resurfaces the knee to restore pre-arthritic anatomy while minimizing soft tissue release (8). Studies comparing outcomes between KA and MA TKAs have reported mixed results, but most recent reviews show superior outcomes (13, 14), especially when trials that include technical/methodological errors are removed, such as the use of personalized cutting blocks instead of robotic technology or not providing the KA algorithm used for implant positioning (15, 16), as restoring moderate to severe alignment deformities increases contact force, implant stress, and bone strain (17). Mid- and long-term survivorship does not appear different between KA and MA (18), with 10-year survival for all-cause revision and aseptic loosening reported as 97.4% and 98.4%, respectively (19). Additionally, stable soft tissue envelopes, acceptable tibial component migrations of less than 0.5 mm, and no associations between joint line obliquity and tibial component migration or soft tissue envelope stability have been reported with KA (20, 21).

The Personalized Alignment technique described in this paper seeks to recreate pre-arthritic anatomy by maintaining patient specific native soft tissue tensions within a range of acceptable alignment boundaries based on the restricted Kinematic Alignment (rKA) principles proposed by Vendittoli et al. (22). Compared to MA, faster recoveries (23), greater satisfaction rates (24), and higher outcome scores (25) have been reported for rKA procedures. On the other hand, personalized TKA requires technologic assistance as standard mechanical instrumentation is likely not accurate enough to achieve patient specific alignment (26). The ROSA^®^ Knee System is a semiactive robotic system that controls the placement of cutting jigs and allows the surgeon to use a preferred saw blade for bone resections and dynamic ligament balancing. Resection depth and cut angle accuracies have been reported as less than 0.7 mm and within 1° (23, 27), respectively, and several studies provide evidence that the ROSA Knee System can improve implant positioning (27), hip-knee-ankle angle (23, 28, 29), and patient reported outcome scores (30) compared to conventional TKA.

The purpose of this surgical technique report is to describe the Personalized Alignment TKA method using the ROSA and Persona knee systems. Our aims are to provide specific procedures for pre-operative planning, intra-operative planning of cuts, resection validation and soft tissue evaluation with robotic-assisted personalized TKA.

## Restricted kinematic alignment boundaries

Computed tomography scans of non-arthritic lower limbs demonstrate a high degree of individual variability in hip-knee-ankle (HKA) angles, with an average absolute deviation of 2.7 ± 2.6° from neutral (31). Osteoarthritic knees present with even greater variability: Almaawi et al. (32) reported nearly 40% of TKA candidates had an HKA greater than 3° with large inter- and intra-individual variabilities in mechanical distal femoral angle (mDFA) and medial proximal tibia angle (mPTA). Recently, classification of the pre-arthritic knee *via* phenotype has been proposed, with the majority (approximately 65%) of non-osteoarthritic knees falling within 177° to 183° aHKA, but only about 18% of knees being comprised of a neutral mDFA and mPTA (33). Because recreating pathological constitutional anatomies of some phenotypes may result in inferior TKA biomechanics and/or abnormal or accelerated wear patterns, Vendittoli (34) proposed alignment boundary restrictions on KA:

• Arithmetic HKA (combined mDFA and mPTA orientation): 0 ± 3°
• Distal Femoral Coronal Alignment: 0 ± 5°
• Proximal Tibial Coronal Alignment: 0 ± 5°

## Pre-operative planning

Pre-operative planning is particularly useful when integrating the ROSA Knee System into practice, though it is not mandatory. The system uses anteroposterior and lateral radiographs to create 3D bone models (X-Atlas^®^, Zimmer Biomet, Warsaw, Indiana) that provide for pre- and intra-operative planning. Distal femoral and tibial cuts can then be planned to recreate the native bone before wear. The accuracy of landmarks on the 3D bone models is within 0.86 and 1.28 mm for the femoral and tibial landmarks, respectively, and the system can predict implant size within ±1 size for the femoral and tibial components in 95.6% and 100% of cases, respectively (35).

Full leg radiographs can also be used for pre-operative interpretation of the patient's constitutional alignment irrespective of image-based planning. The lateral distal femoral angle, medial proximal tibial angle and posterior tibial slope are preliminarily measured to determine constitutional alignment at this stage. These values will then be compared to intra-operatively acquired references to adjust the bone cut. Although cartilage loss will not affect bone cut planning on x-Rays, landmarks should be taken on areas of bone without major erosion.

## Exposure

Any standard exposure may be used. Care should be taken to avoid soft tissue releases usually performed with wide medial exposure and the deep medial collateral ligament (MCL) attachment should be preserved. Removal of the osteophytes is not required at this stage.

## Landmarking and reference for cuts

Prior to landmarking and bone registration, femoral and tibial rigid bone references are installed.

### Femoral landmarking

**Figure 1:**
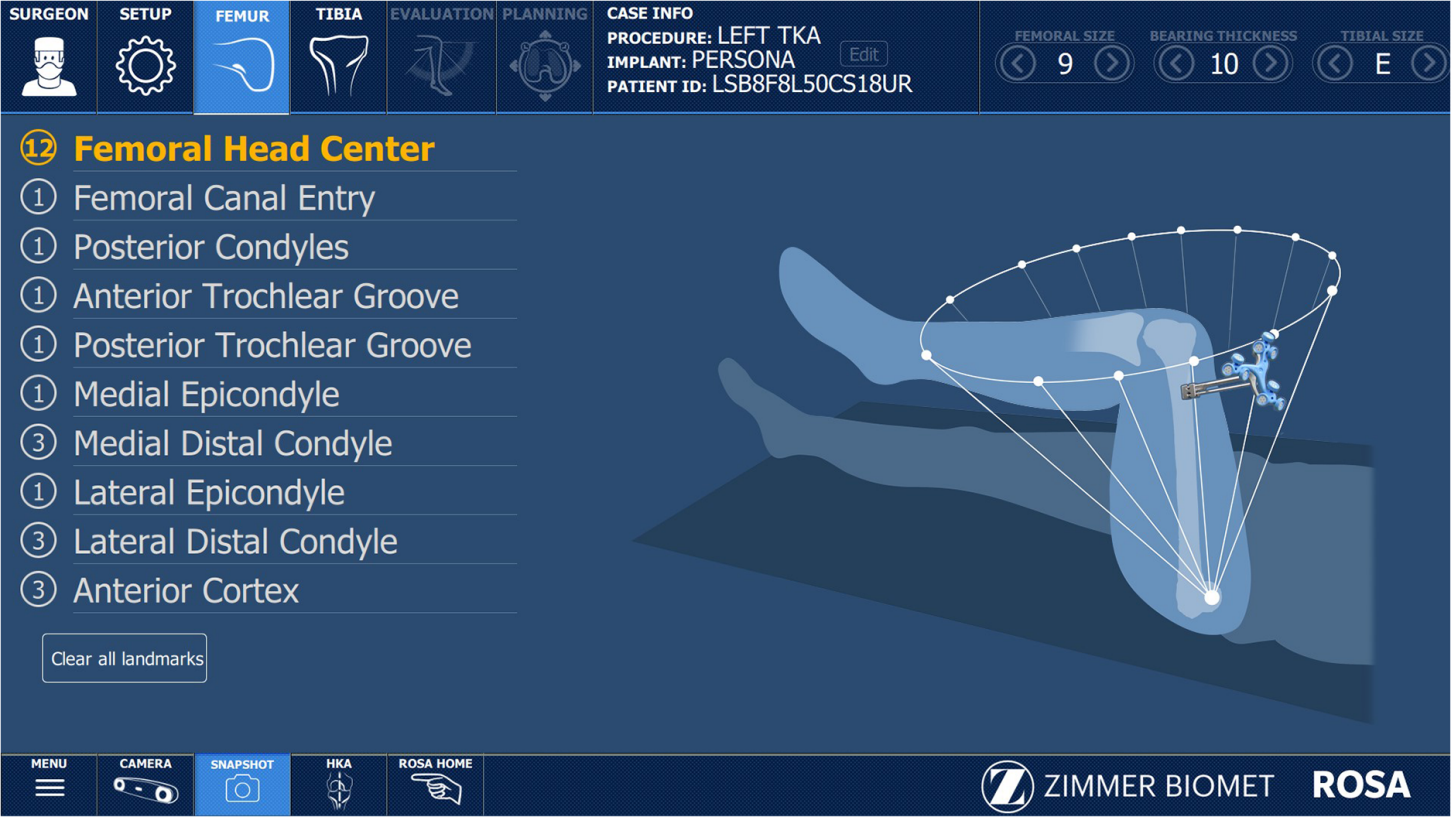
Registration screen displaying the workflow for the required femoral anatomic landmarks.

Femoral head center (Figure 1), femoral canal entry point, anterior and posterior trochlear groove, and the medial and lateral epicondyle landmarks are taken according to the ROSA Knee User Manual. When registering the joint surfaces, it is important to acknowledge that human knee cartilage thickness varies from 2 to 3 mm (36).

The posterior condylar axis (PCA) point is determined with the Posterior Condyle Digitizer. In Personalized Alignment, the rotation of the femoral component is set based on the native posterior femoral condylar surface and is usually 0° of rotation with reference to the PCA unless focal wear is present on one of the condyle surfaces. While cartilage cannot be pierced with the posterior condyle digitizer, significant wear should be noted.

**Figure 2:**
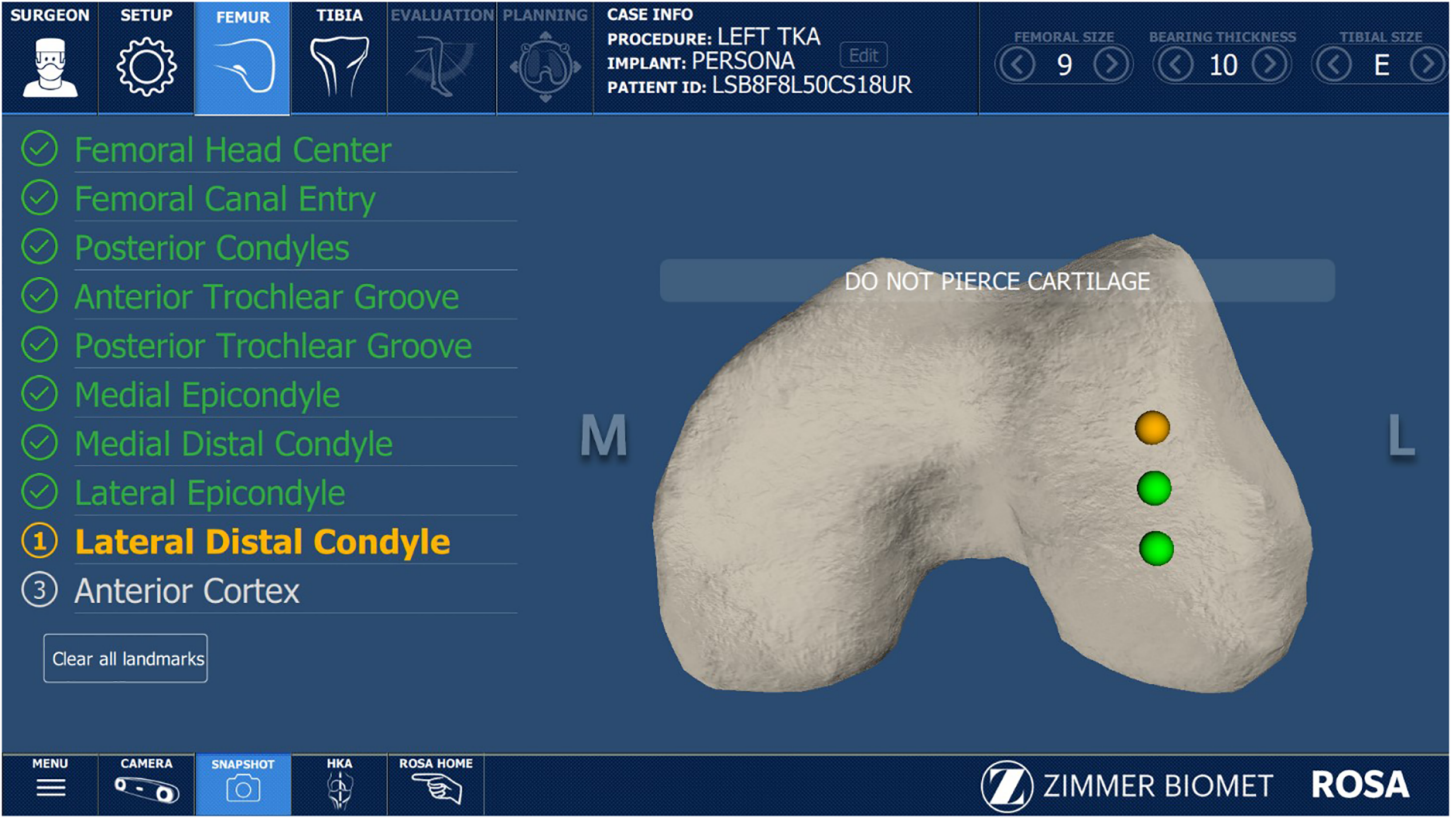
Registration screen displaying lateral distal condyle landmarks.

**Figure 3:**
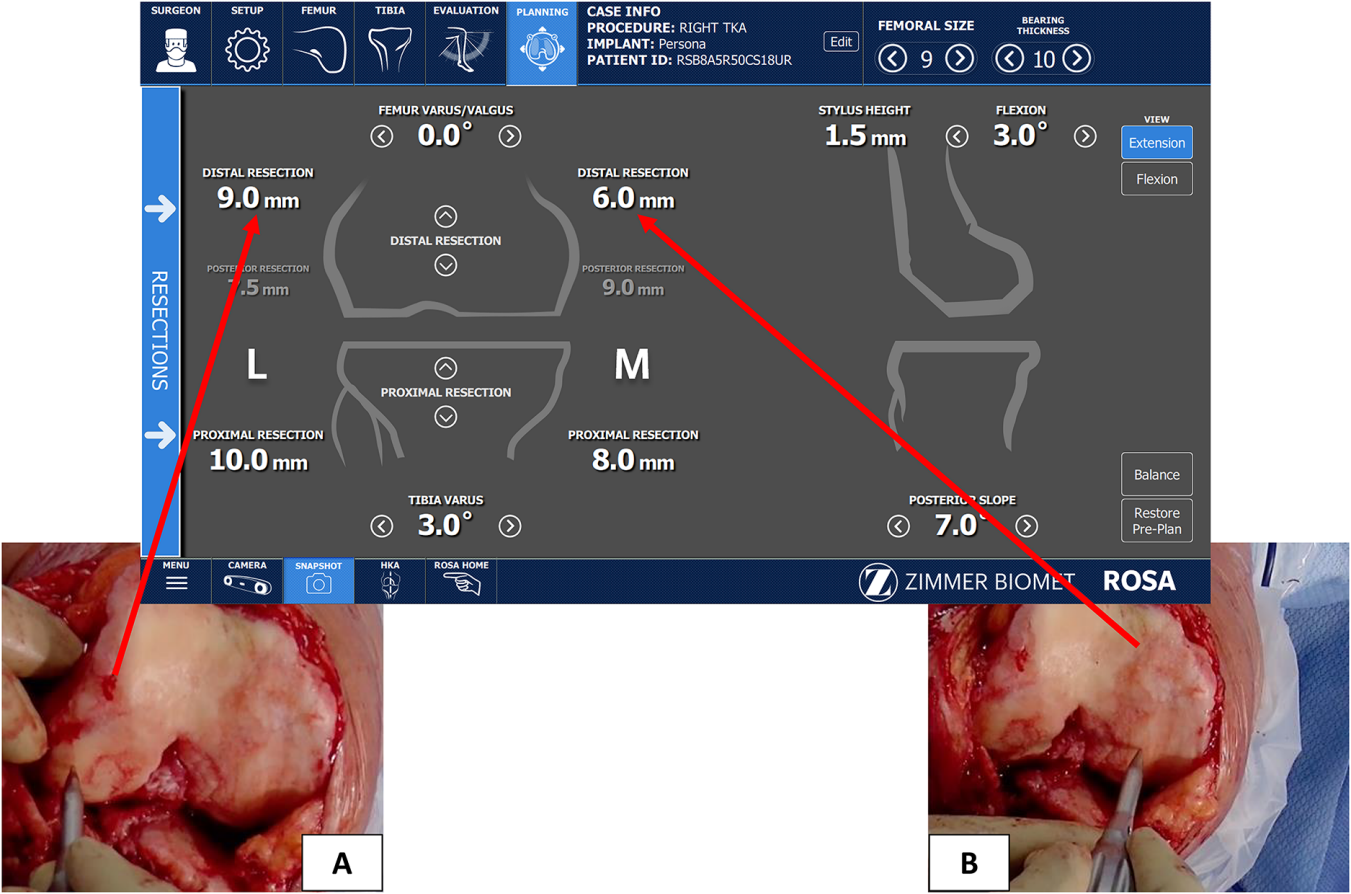
The lateral condyle (**A**) illustrates landmarking on Normal cartilage while the medial condyle (**B**) is landmarking on subchondral bone where there is both 2 mm cartilage loss and small (∼1 mm) bone erosion. The registration screen displays the planned difference in registered levels, and the plan deducted the bone loss from the values.

Next, the medial and lateral distal condyles will be landmarked by obtaining three points on the distal surfaces of the respective condyles with the Registration Pointer (Figure 2). If the bone is exposed, the landmarks will be directly on the bone and any erosion should be evaluated and subtracted from the cut. If cartilage is still present, one of the following two tactics may be taken (Figure 3):

1. Do not pierce the cartilage and instead set the level of resection so that the articular surface of the component matches the native cartilage.
2. You may expose normal bone and take landmarks on subchondral bone, taking care to avoid bone defects.

Tactic 2 is our preferred technique because it is more accurate to use bony landmarks as reference on both sides than to estimate the thickness of cartilage lost, especially in cases of partial wear.

Finally, the anterior femoral cortex is determined by obtaining three points at the middle of the anterior surface of the distal femur. As previously discussed, no rotation of the femoral component is required (true resurfacing) and therefore the anterior cortex landmarks do not need to be taken as lateral as with mechanical alignment and it's standard 3° of external rotation.

### Tibial landmarking

The ROSA Knee User Manual should be followed for the medial and lateral malleoli, medial third of the tibial tubercle, tibial canal entry point, and PCL insertion point. Regarding landmarking for the medial and lateral plateau resection, first obtain a point at the middle of the medial and lateral tibial plateau with the Registration Pointer, taking care to select medial and lateral points at equal distances antero-posteriorly to account for the tibial slope. Landmarks may be taken on cartilage or on exposed normal bone. Significant erosion is often localized, and thus landmarks should be taken in a zone without bone loss, if possible. If not possible, any bone loss thickness should be estimated and deducted from the cut.

## Initial evaluation

**Figure 4:**
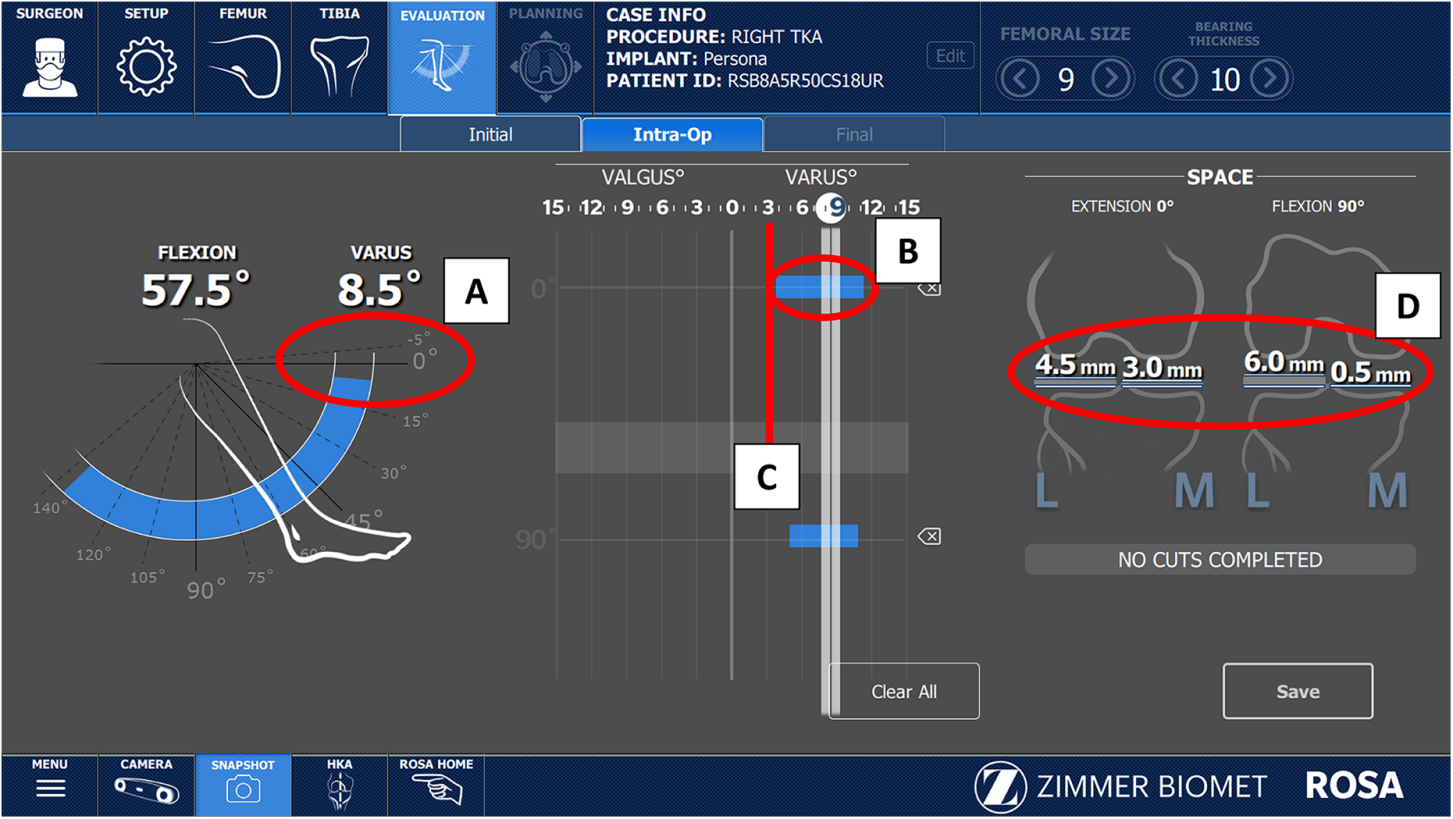
(**A**) Initial knee range of motion showing fixed flexion contracture before any release. Note that no adjustment in thickness are made in the distal femoral cut for fixed flexion deformity, since posterior osteophyte removal almost always corrects this deformation. Results of the varus/valgus stress test used to assess patient laxity in extension (**B**) and flexion. The maximal varus correction in extension (**C**), and the maximum laxity in mm (**D**).

Our next step in the workflow is the evaluation of maximum extension and flexion and the maximum possible correction in extension. There is no need to remove osteophytes before this step either. Valgus and varus stress tests are applied at 10 and 90° to measure the degree of distraction in the medial and lateral compartment and are used by the ROSA Knee System to quantify laxity in each compartment. We use this information to check for maximal deformity correction (red line in Figure 4) and to examine the patient's native ligament tension and screen for pathological hyperlaxity. This stressed HKA evaluation has been shown to correlate well with the arithmetic HKA and can help the surgeon set their HKA goal (37). In our Personal Alignment philosophy, we want to preserve the native laxity by protecting the patient's soft tissue envelope. Compared to the medial compartment, laxity in the lateral compartment of the native knee has been reported as 0–2 mm more laxity in extension and 1–4 mm more laxity in flexion. Additionally, the medial compartment is expected to have 1–2 mm more laxity in flexion than extension (11). At this stage, the arthritic compartment may be considerably tighter as a result of osteophytes, and the variation from medial to lateral may not be assessable.When the lateral compartment is intact (varus knee), the patient's laxity in the lateral compartment will differ from flexion to extension, and is usually looser in flexion (2 mm or more in our case). The Personalized Alignment technique should recreate this native laxity in the flexion lateral compartment which will allow for medial pivot and normal kinematics. Surgeons new to this technique may evaluate their patient's lateral compartment laxity at this stage to compare with the final evaluation to ensure recreation of the soft tissue envelope.

At this stage the gaps will change after bone and osteophyte resection, so we do not consider them in this stage in the planning process.

## Intra-operative planning and adjustment

Given that evaluation of the degree of cartilage wear during landmarking is subjective and native cartilage thickness is slightly variable (2–3 mm), we recommend landmarking on exposed normal bone when possible to more accurately estimate the patient's native femoral and tibial mechanical axis and plan resections. If the landmark was taken on exposed normal bone on the medial and lateral sides of the femur and tibia, plan to resect 8 mm (equal to the 10 mm implant thickness when added to the 2 mm of lost cartilage) from the distal femur and proximal tibia. If bone erosion is not present, adjustment of the cuts to resurface the native bone is done by modifying the varus/valgus angle of the distal femoral and tibial cuts until the values of resection are equal medially and laterally, and then changing the cut thickness until it is 7–8 mm throughout. When significant bone erosion is present without an intact area, deduct the bone loss from the planned cut thickness. For example (Figure 3), a medial tibial plateau with 2 mm bony erosion should have a 5–6 mm cut (2–3 mm missing cartilage +2 mm bony erosion +6 mm cut = 10 mm for component thickness).

### Varus/valgus adjustment

**Figure 5:**
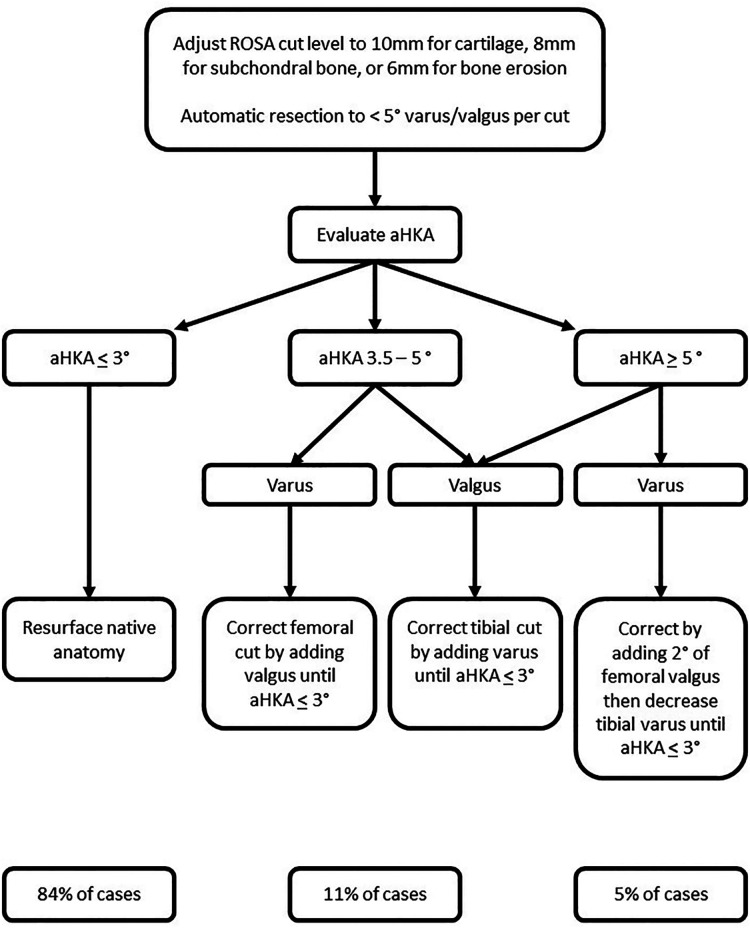
Restricted kinematic alignment algorithm for personalized total knee arthroplasty. For example, a patient with an aHKA of 8° varus (2° femoral and 6° tibial varus) we will first modify the tibia to 5°. Next, we will reduce the femoral angle by 2° to 0°. Finally, we reduce the tibial angle to 3° to produce an aHKA of 3° varus. With a valgus knee, the femur is often the main contributor and will be adjusted first. For example, a patient with and aHKA of 9° valgus (8° femoral and 1° tibial valgus) we would first modify the femur to 5° producing a valgus aHKA of 6°. We will then modify the tibia by 3°, bringing it to 2° of varus, thus producing an aHKA of 3° valgus.

In an analysis of 4,884 computed tomography knee scans, approximately 51% fell within the rKA limits (mDFA and mPTA ≤ 5° and aHKA ± 3° of neutral) and required only resurfacing of the femoral and tibial articular surfaces for KA TKA (32). In this same study, approximately 33% of cases presented with either mDFA or mPTA greater than 5° which could be corrected to within rKA limit by modifying the abnormal angle to within 5°. Cumulatively, these cases (approximately 84% of cases) will be automatically taken care of by the ROSA Knee System since it's not possible to set the varus-valgus cut at greater than 5°. Approximately 11% of cases will require only minor adjustment (<2°) for the HKA to fall within the limit of rKA, and these cases rarely require soft tissue adjustment. Finally, about 5% of cases present with extreme abnormalities (tibial and femoral anatomy contributing in the same direction to the aHKA deviation) that required significant modifications to be brought back within rKA limits. In these extreme cases, the rKA personalized philosophy is to minimize femoral modification as this is thought to have major effects on knee kinematics (38). These extreme deformity cases often require some soft tissue release to obtain adequate final balance. Figure 5 presents an algorithm related to rKA planning, according to Vendittoli (22) principles.

There are two main points that should be considered when modifying cuts to fit within the rKA boundaries. First, when making adjustments, cuts on the intact side (lateral for varus and medial for valgus) should never be more than 8 mm on a bony reference or 10 mm on normal cartilage. Using the unworn condyle as a reference prevents over-resection, and though rarely needed, a recut may be easily performed.

Second, ligamentous releases are to be expected when anatomy correction exceeds 2–3°. If the medial compartment is too tight in a varus knee, often a deep MCL release will open the gap wide enough to match the implant thickness. Releases are usually less extensive than a similar case done with mechanical alignment. Similarly, conservative pie crusting will address a tight lateral compartment in the valgus knee.

### Femoral rotation

If cartilage is present, the surgeon should plan to resect 9 mm equally from both posterior condyles, with the objective to resurface the pre-arthritic knee such that most patients will have a neutral rotation in reference to the PCA. The transepicondylar axis is not generally used to set the rotation in the Personalized Alignment technique because it does not help to recreate the pre-arthritic anatomy over the PCA, is highly variable (39), and the resections planned in the ROSA Knee System are based upon femoral landmarking of the posterior condyles. If full cartilage wear is present on one or both condyles, the cut should be adjusted to resect less, to match the posterior thickness of the implant system. Although rare for the medial side, it's more frequent for valgus knees with posterolateral osteoarthritis. For those cases 9 mm should be cut from the medial side and 7–8 mm from the lateral side, thus creating mild external rotation in relation to the present PCA, but neutral relative to the pre-arthritic knee.

### Femoral flexion and tibial slope

Femoral flexion and tibial slope also display significant inter-individual variation; however, it is not clear what implications this has on knee kinematics or outcomes. At this time, there does not appear to be a clear “safe range” for femoral flexion established in the literature. There may be small ROM benefits (40, 41) to increasing femoral flexion beyond 5°, but these may be offset by an increased risk of flexion contracture (42) and worsening PROMs, especially with flexion in excess of 8° (43). To match the native diaphyseal femoral bowing, femoral flexion should normally be around 3°, though this can be adjusted according to the patient's specific anatomy and femoral notching can be avoided by mildly increasing the degree of flexion up to 5°.

In most cases we set the tibial slope to 5° posteriorly. We prefer to use the Medial Congruent (MC) implant, which stabilizes the knee in flexion and extension, allowing the surgeon's preference to dictate whether to retain or sacrifice the PCL. On the other hand, releasing the PCL is known to increase the flexion space and may require compensation by over-resection of the distal femur and joint line modification. In all cases, the PCL should not restrain the knee. If the flexion space is tighter than the extension space, the PCL should either be sacrificed, or the tibial slope increased (with confirmation that all osteophytes have been removed prior to assessing gaps).

### Balance tool

**Figure 6:**
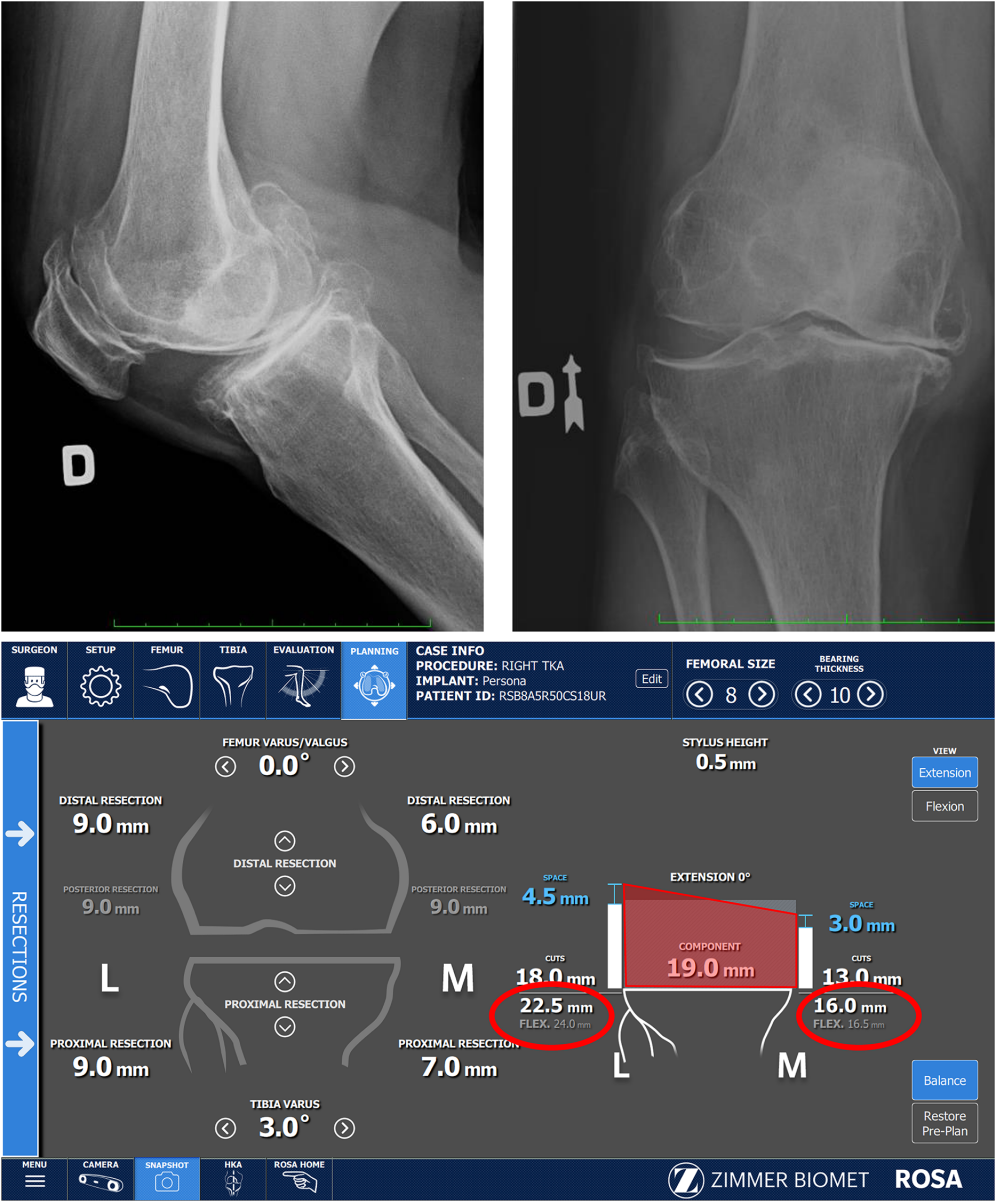
Large posterior osteophyte present in x-ray, explaining flexion deformity. Medial osteophytes still present, as seen in the x-Ray, are reflected on the registration screen as inadequate space in the medial compartment (16 mm of space compared to 19 mm component thickness).

ROSA will provide gap analysis throughout the workflow *via* the Balance Tool, and once the resection plan has been input, it will show the level of resection, component thickness, and gaps available after component implantation. Compared to a functional technique, we do not consider these initial gaps in our planning, nor do we use them to try to balance the knee. Osteophytes have not been removed yet and following the cut, the worn side will display a larger gap. Instead, the Balance Tool should be predominantly used to verify if the unworn surface has sufficient resection to accommodate the component during the intra-operative planning phase. For example, when doing a personalized TKA for a varus knee, greater laxity is to be expected in the lateral compartment in extension and flexion, however, sometimes it will appear the medial compartment is too tight because the osteophytes have not yet been removed (Figure 6). For most cases this will correct itself after the cuts have been made and osteophytes removed.

## Performing and validating the cuts

The order of resection for our personalized knee alignment is distal femur, followed by the proximal tibia and finally the posterior femur. For the distal femur, all retractors should be positioned prior to moving the robotic arm and cutting block into the cutting plane. We prefer to use a curved Hohmann Retractor on the side of the robotic arm and straight retractor on the camera side. As the robotic arm approaches the joint, the robot will enter a “collaborative” mode and the surgeon then moves the arm into its final position. The live cut screen can then be used to verify the medial and lateral resection levels compared to the planned levels, and the positioning for the cutting block. Once the cutting block is in the accepted position, the surgeon places two pins to secure it in place. At this stage it is important to avoid any movement of the patient's leg or any change in retraction. Most of the cuts will be just under the level of the osteophytes and will remove them with the resection, however, any residual osteophytes can easily be removed after the resection. New adopters may verify the thickness of the resected bone stacked on top the saw blade independently with a caliper.

Validation should be performed after the distal femoral and proximal tibial cuts with the verification tool to confirm the bony resection levels. If the validated varus/valgus angle is different than the planned angle, the cut guide can be brought back to make sure the cut was complete and adequate, or the plan can be adjusted to ensure the final HKA angle is within 3°of neutral (for example: slight varus correction of the tibial cut after validation of the femoral cut at 1.5° valgus instead of 0.5° valgus). The final validated femoral flexion should be dialed in to the planner since this could change the femoral component sizing and the risk of notching.

## Soft tissue evaluations and implantation

We first assess ligamentous tension and fit with trial components. Range-of-motion (ROM), lateral and medial stability in extension and flexion, and patella tracking can be verified before final implantation. A loss of full extension is indicative of too much joint tightness and requires either decreasing polyethylene thickness, posterior release, or recutting the distal femur. Tibial plateau lift-off in flexion is also indicative of excessive tightness in flexion and may require either PCL release, increased tibial slope or downsizing the femoral component.

A major objective of this Personalized Alignment technique is to reproduce the patient's pre-arthritic ligament tensions. Collateral ligament laxity in a healthy, native knee are not isometric through the arc of flexion (12). We normally expect a small difference of 1–2 mm between the medial (1–2 mm) and lateral (2–3 mm) compartment openings at 10° degrees of flexion, and laxity increases with greater degrees of flexion in the lateral compartment, especially with PCL resection (Table 1) (11). The initial evaluation can be used to examine the constitutionally greater laxity of the lateral component in flexion, with lateral gap values usually ranging between 3 and 7 mm.

**Table 1:** Flexion and extension gaps in the rKA knee.

	Medial	Lateral	
		PCL Retained	PCL Resected
Extension	1–2 mm	1–3 mm	1–4 mm
Flexion	1.5–3 mm	3–7 mm	3–8 mm

The small difference in laxity in extension provides stability during initial contact of the stance phase of the gait cycle (44), and medial stability combined with lateral laxity during flexion allows for pivoting around the medial compartment as seen in the native knee. We therefore recommend a MC bearing in this procedure. This bearing type provides enhanced stability against medial anteroposterior translation with greater lateral compartment anteroposterior articulation (45). Thus, the Personalized Alignment technique and a MC component work together to stabilize the knee medially while allowing movement and rotation laterally (46), recreating normal kinematics.

The Personalized Alignment technique minimizes the need for ligamentous releases. However, since pathological anatomies are not recreated, some cases may still require minimal adjustment, especially in the 5% of cases where a significant correction may be performed. Releases performed in Personalized Alignment will usually be minimal compared to the extent of release necessary for mechanical alignment, but must still ensure the component has sufficient space in the unaffected compartment. We should note that while the gaps obtained during the soft tissue evaluations may be used as a guide for releases, high inter-individual variabilities in the force and technique should also be considered. Surgeon experience of gap appreciation is still important and should prevail, acknowledging that we are aiming for asymmetrical gaps as explained earlier.

## Expected outcomes

Recreating the patient's pre-anthric knee contour and preserving the patient's native joint line, plane of flexion and soft tissue envelope helps to restore the native kinematics of the knee. When combined with a modern medial congruent tibial implant, we believe the Personalized Alignment technique may improve patient satisfaction and clinical outcomes following TKA. A large meta-analysis of 1,112 cases reported greater Western Ontario and McMaster Universities Osteoarthritis (WOMAC) scores, Knee Society Score (KSS), flexion ROM, and walking distance for the KA technique compared to mechanical alignment (14), and a recent clinical retrospective study reported improved knee and function KSS scores at 6 months with a Personalized Alignment technique compared to non-robotic mechanically aligned TKA (26). Regarding the MC bearing, a meta-analysis (47) reported similar WOMAC and KSS, but improved forgotten joint scores with the MC bearing, and clinical studies have shown improved pain, satisfaction, and ROM with the MC compared to posterior stabilized components (48, 49). Of particular importance to the Personalized Alignment technique, a recent study reported better 2-year ROM (8°), KSS pain scores (8 points), and forgotten joint scores (10 points) when performing KA with a medial-stabilized compared to a posterior stabilized component (50). Preliminary data from a single surgeon study also suggests better Knee Injury and Osteoarthritis Outcome Scores (KOOS) for quality of life and forgotten joint scores at 1-year follow-up with a medial-stabilized component compared to a cruciate retaining design (51) with rKA, while a greater incidence of tibial implant loosening and lower survival have been reported when a posterior stabilized component was used in rKA (52).

## Conclusion

Mechanical alignment has been the most reliable way to execute a TKA when only conventional instrumentation was available to surgeons. While mechanical alignment creates a standard, but non-anatomical position, it can require a multitude of corrections during the surgery, and achieving a neutral mechanical alignment (180 ± 1°) is not associated with greater functional outcomes, ROM, or prothesis longevity (10). Emerging evidence suggests Personalized Alignment in conjunction with a MC implant may result in a faster recovery of function and improved clinical outcomes (53).

To perform the Personalized Alignment technique, accurate and precise tools are necessary to reliably recreate patient anatomy and ensure components are not implanted in a position that may compromise long term outcomes. Robotic assisted surgery is gaining in popularity and may be the future of orthopedic surgery. The ROSA^®^ Knee System provides accurate, precise, and efficient assistance allowing the surgeon to perform the Personalized Alignment techniques in TKA.

While the ROSA^®^ Knee System is a valuable technology, surgeons will only achieve their full potential for improving patient outcomes and restoring quality of life when old dogmas are challenged. This Personalized Alignment technique for TKA with the robotic system described may act as a mechanism to move the field beyond traditional mechanically aligned TKA.

## Data Availability

The original contributions presented in the study are included in the article/Supplementary Material, further inquiries can be directed to the corresponding author/s.
